# Evaluation of Clinical and Histopathological Diagnosis of Kaposi’s Sarcoma at Muhimbili National Hospital, Dar es Salaam, Tanzania

**DOI:** 10.24248/eahrj.v8i3.809

**Published:** 2025-01-30

**Authors:** Edrick M Elias, Amos Rodger Mwakigonja

**Affiliations:** a Department of Pathology, Catholic University of Health and Allied Sciences, Mwanza, Tanzania; b Department of Anatomical Pathology, Muhimbili University of Health and Allied Sciences, Dar es Salaam, Tanzania.

## Abstract

**Background:**

Treatment and outcome of Kaposi’s sarcoma (KS) depend on a correct histopathological diagnosis, however, most KS cases in developing countries are diagnosed clinically without histopathological confirmation, which results in either over or under-diagnosis. Also, due to the number of histopathological mimickers in different stages of KS which include benign to fatal conditions, the histopathological diagnosis of KS is not always correct. However, the HHV-8-LANA-1 Immunohistochemical (IHC) stain is positive in nearly all KS lesions and is considered to be an important diagnostic tool to differentiate KS from its histological mimickers. This study aimed to determine the quality of Kaposi’s sarcoma diagnosis at MNH and whether it can be improved by the routine of HHV-8-LANA-1 immunohistochemical stain.

**Methodology:**

This was a retrospective cross-sectional hospital-based study of all KS cases diagnosed either by clinical, histopathological, or both in 2018. KS was diagnosed based on H&E morphology and confirmed by HHV-8-LANA-1 immunohistochemistry. The diagnosis utility of clinical and histopathology was compared with HHV-8-LANA-1 immunohistochemistry.

**Results:**

There was almost perfect agreement between initial and reviewed histopathology for KS diagnosis (Kappa value= 0.892, *p-value=.000*). The clinical diagnosis concordance rate was 61% with no agreement (Kappa value −0.123, *p-value=0.102*). Clinical differential diagnosis included a wide range of pathological conditions ranging from less severe inflammatory to fatal malignant conditions. There was a substantial agreement between initial histopathology and HHV-8-LANA-1 IHC for KS diagnosis (Kappa=0.70, *p-value* .000) with a histopathology concordance rate of 88%.

**Conclusion:**

Histopathological examination of all clinical KS suspicions and HHV-8-LANA-1 immunohistochemistry confirmation is required since the study showed that the histopathology misdiagnosis of KS at MNH was unlikely to be the result of human error. We recommend that in every clinically suspected KS case, an adequate tissue biopsy should be taken for histopathology analysis and HHV8-LANA-1 immunostaining to avoid inappropriate treatment.

## BACKGROUND

Kaposi’s sarcoma (KS) is a systemic multifocal angiomatous tumor that expresses both vascular and lymphatic endothelial markers by Immunohistochemistry (IHC).^1^ Clinically, it is characterized by multiple red to purple macular to papular, slowly growing lesions. The tumor is associated with infection by KS-associated herpes virus (KSHV).^2^ Epidemiologically, it is classified as Classical KS, Endemic African KS, and Iatrogenic KS secondary to immunosuppression in organ transplant patients and acquired immunodeficiency syndrome (AIDS) related KS. Nevertheless, the increased incidence of human immunodeficiency virus (HIV) in Africa makes it both clinically and histologically difficult to distinguish Endemic African KS and AIDS-related KS in people living with HIV.^3^ Despite the global decrease in KS incidence, AIDS-related KS is still prevalent in several Southern and Eastern African countries and is estimated to be the leading cause of all cancer mortality in 2018.^4^ Despite the significant decline in HIV incidence in Tanzania (estimated to be 4.6% overall, in urban setting the incidence is still high which is 7.5%.^5^ This situation contributes to persistence of AIDS-related KS. Tanzania is a sub-Saharan Africa country in the KS belt, presumed to have a high incidence of KS, but due to the absence of a population-based cancer registry, reliable data on KS incidence is lacking. KS in Tanzania is estimated to rank among the top four cancers.^4^ Hospital-based studies in Tanzania have shown incidence between 2.4% to 12%.^6–9^ Diagnosis of KS is not straightforward both clinically and histologically due to other lesions that mimic KS ranging from benign to life-threatening aggressive conditions.^10–12^

The clinical differential diagnosis for KS depending on the stage, includes warts and dermatofibroma, acute or life-threatening conditions such as bacillary angiomatosis, lymphoma, squamous cell carcinoma and other nonneoplastic conditions such as psoriasis, sarcoidosis and tuberculosis.^10–14^ The histopathological diagnostic challenges and KS mimickers also depend on pathological stages. The patch stage represents early lesion in KS development, and this is the most challenging stage in histological diagnosis. Early histologic changes which include mild inflammatory infiltrates, newly formed slit-like or jagged vascular spaces, and promontory sign may be inconspicuous and for that reason, it can be missed on biopsy.^15,16^

The histopathology differential diagnosis patch and plagues KS stages include chronic inflammation, granulation tissue, vascular anomalies, tufted angioma, targetoid hemosiderotic hemangioma, cavernous hemangioma, and acroangiodermatitis.^15,17,18^

In the nodular stage, histopathology diagnostic features are well established and thus pose fewer diagnostic challenges, but sometimes the differential diagnoses in this stage include; angiosarcoma, spindle cell hemangioma, dermatofibroma, dermatofibrosarcoma protuberans, and pyogenic granuloma.^15^ In a study done in South Africa, when clinical suspicion of KS was evaluated against histopathology and Human herpes virus 8 latent nuclear antigen 1 (HHV8-LANA-1) immunohistochemical stain clinical discordant rate was 30.2%, and in the same study, histology when compared with HHV8-LANA-1 immunohistochemical staining the histology discordant rate was 9.2%.^12^ Also, in a study by Erin Amerson et al, in Kenya and Uganda among people living with HIV, the clinical positive predicted value was 77% and the discordant rate between East African Pathologists and United States of America-based Dermatopathologists was 31%, sensitivity and specificity of 68% and 89%, respectively.^10^ HHV-8-LANA-1 is expressed up to 100% in KS lesion biopsies and detection of HHV8 on tissue is diagnostic for Kaposi’s sarcoma,^11,19–23^ which is a readily available and cost-effective method to confirm KS diagnosis. No difference in the detection of KS-associated virus on tissue between HHV-8-LANA-1 immunohistochemistry and the molecular techniques.^24,25^

The HHV-8-LANA-1 immunohistochemical confirmation of KS was not routine at Muhimbili National Hospital (MNH). To the best of our knowledge, there is no published information regarding the clinical and histological diagnostic accuracy of KS at MNH. This study was helpful in determining whether routine KS diagnosis would be improved by the use of HHV-8-LANA-1 immunohistochemical stain.

## MATERIALS AND METHODS

### Study Setting

This study was conducted at MNH, Central Pathology Laboratory (MNH CPL). MNH is a National Referral Hospital, Research Center, and MUHAS teaching hospital located in Dar es Salaam, Tanzania. There are Dermatologists in the department of internal medicine at MNH.

The CPL receives biopsies from different health facilities in Tanzania. KS is diagnosed at MNH mainly through H&E-stained histomorphology evaluation. HHV-8 LANA-1 immunostaining is not routinely done. Most Pathologists at MNH are general Pathologists, except one who is a Dermatopathologist.

### Study Design and Duration

This was a one-year retrospective cross-sectional hospital-based study.

### Study population

All KS cases were diagnosed either by clinical, histopathological, or both from 1^st^ January to 31st December 2018.

### Inclusion and Non-Inclusion

The study included all KS cases with clinical and/or histopathology diagnosis of KS and it excluded cases whose tissue blocks could not be retrieved for subsequent HHV-8-LANA-1 immunohistochemical stains and those whose tissue quality was poor for histomorphology and immunohistochemical evaluation.

### Sampling and Sampling

All 130 KS cases diagnosed in the year 2018 were included in the study.

### Data collection

Clinical parameters were retrieved from histopathology request forms; histopathology diagnosis was also confirmed in the hospital laboratory system. Hematoxylin and eosin (H&E) stained slides and tissue blocks were retrieved from the archive and missing slides were re-stained.

#### Hematoxylin and Eosin Staining

The formalin fixed paraffin embedded tissue blocks were sectioned by using a manual microtomy set at four micrometers, the tissue ribbons were placed in a warm water bath at 50°C and then placed in a glass slide to make a tissue section. The tissue sections were drained and placed in a hot plate at 60°C for 30 minutes for dewaxing. The tissue sections were then placed in two changes of xylene each for 10 minutes to remove paraffin wax. The tissue sections were rehydrated in descending concentrations of ethyl alcohol, from absolute alcohol to tap water. The tissue sections were stained in Harris hematoxylin for 10 minutes. The stained tissue sections were rinsed in tap water and then differentiated by using 1% acid alcohol for 10 seconds. The tissue section was rinsed in tap water and then bluing was done using running tap water for 10 minutes. The tissue sections were then counterstained in 1% aqueous eosin for 3 minutes and then rinsed in tap water, followed by dehydration through ascending concentrations of ethyl alcohol from 95% to absolute alcohol. Tissue clearing was done through two changes of xylene for 5 minutes each and then mounted with a semi-automated coverslipping machine.

#### Immunohistochemistry (IHC) Staining and Evaluation

Four-micrometer tissue sections from formalin-fixed paraffin-embedded (FFPE placed on positively charged glass slides were examined immunohistochemically using mouse ant-HHV-8-LANA-1 monoclonal antibody (clone 13B10) batch number 05269229001 ready to use.

Tissue sections were stained with automated immunostainer (Ventana GX) using heat-induced epitope retrieval by Tris buffer, PH 7.6 for 32 minutes, and OptiView peroxidase inhibitor was applied for 4 minutes to block endogenous peroxidase activity. The primary antibody was incubated for 32 minutes. Detection was by Ventana OptiView DAB detection kit which was composed of HQ universal linker incubated for 8 minutes, OptiView HRP Multimer incubated for 8 minutes, OptiView H2O2 and DAB incubated for 8 minutes followed by OptiView copper incubated for 4 minutes. Hematoxylin II was incubated for 12 minutes for counterstaining followed by a bluing agent and coverslipping. A piece of the known case of KS was mounted on every test slide as a positive control and an internal negative control was used.

Strong, diffuse, nuclear staining in >10% of tumor cells was considered HHV-8-LANA-1 positive results. In all cases, positive control stained positive. The interpretation was done by two independent pathologists.

### Data Analysis

Data were recorded and analyzed in SPSS version 20. Age and duration of symptoms were summarized in range and median. Kappa was used to measure interrater reliability between two variables and a value above 0.00 was considered as an agreement. Concordance rate, positive predictive value, and sensitivity were calculated between clinical KS diagnosis against HHV-8-LANA-1 IHC, and initial histopathology diagnosis against HHV-8-LANA-1 IHC. Discordances were further classified as a false positive or false negative and categorized as inflammatory, benign, and malignant. The results were compared with the findings from the literature; reasons for differences or similarities were discussed. Also, recommendations on useful good practices were proposed.

### Ethical Review and Approval

Ethical clearance to conduct this study was obtained from the Muhimbili University of Health and Allied Sciences (MUHAS) Institutional Review Board IRB) (Ref no: DA287/298/01A) and permission to conduct the study was obtained from the Executive Director of MNH through the Head of the Histopathology unit.

Patients’ information was strictly kept confidential; no patient names or other clinical information were revealed to any person not involved in this research. This study did not have any adverse effects on the subject’s health. Research identity code numbers were used instead of the names of patients.

## RESULTS

One hundred thirty (130) patients were recruited; 5 cases were excluded because tissue blocks could not be traced in 3 cases and 2 cases were inadequate tissue biopsy for interpretation. The majority were male 71(56.8%), age range of 9–86 years with a median age of 41±15.5; HIV status was not reported in 47(37.6%), and HIV positive 62(49.6%), on ART 46(74.2%). Lesions were mainly cutaneous 74(75.2%) with a median symptom duration of 6 months, and lower limbs were the most biopsied site 67(53.6%). Among 125 cases, 88(70%) were HHV-8-LANA-1 IHC confirmed KS, and 9 (10.2%) among confirmed KS cases were HIV negative and classified as Endemic African KS. There was almost perfect agreement between initial and reviewed histopathology for KS diagnosis (Kappa value= 0.892, *p-value= 0.000*) (Table 1). There was no agreement between clinical and HHV-8-LANA-1 IHC for KS diagnosis (kappa −0.123, p-value=0.102) with a clinical diagnosis concordance rate of 61% and a positive predictive value of 67.5% (Table 1).

**Table 1.** Summary of Agreement between Initial and Reviewed Histopathology, Concordance Rate between Clinical and HHV-8-LANA-1 Immunohistochemistry (IHC), and Concordance Rate between Initial Histopathology and HHV-8-LANA-1 IHC for KS Diagnosis

Analysis	Kappa value	P-Value	Concordance rate (%)	PPV (%)
Initial against reviewed histopathology diagnosis	0.892	0.000	-	-
Clinical diagnosis against HHV-8-LANA-1 IHC	−0.123	0.102	61	67.5
Initial histopathology diagnosis against HHV-8-LANA-1 IHC	0.70	0.00	88	88.4

**Figure 1.**
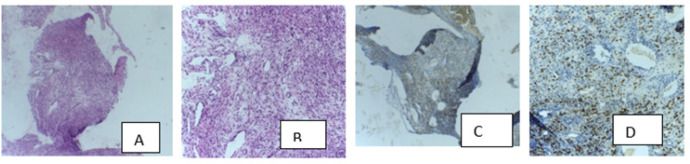
Concordant Pair of Histopathology and Positive HHV-8-LANA-1 IHC in a Rare Case of Nasopharyngeal Kaposi’s Sarcoma

**Figure 2.**
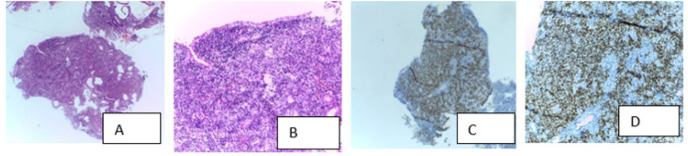
Concordant Pair between Histopathology and Positive HHV-8-LANA-1 IHC in Case of Gastric Kaposi’s Sarcoma in a Case of Gastric Kaposi’s Sarcoma

Clinically KS was wrongly diagnosed in 35 cases, and it was missed 15 cases which include a wide range of pathological conditions ranging from less severe inflammatory to fatal malignant conditions (Table 2). Also, there was a substantial agreement between initial histopathology and HHV-8-LANA-1 IHC for KS diagnosis (Kappa=0.70, *p-value 0.000*) with a histopathology concordance rate of 88% and a positive predictive value of 88.4% (Table 1). Pyogenic granuloma was the most common differential diagnosis difficult to differentiate from KS (Table 3). This study also demonstrated a rare case of nasopharyngeal KS (Figure 1) and gastric KS (Figure 2).

**Table 2.** Clinical KS Diagnosis Discordance before Histopathology or HHV-8-LANA-1IHC

	False positive	False-negative	(N=48)
Malignant	Cutaneous T cell lymphoma (1)	Lymphoma (1)	2
	Burkitt’s Lymphoma (1)	Recurrent sarcoma (2)	3
	Large B cell lymphoma (1)	Rhabdomyosarcoma (2)	3
	DFSP (2)	Leiomyosarcoma (1)	3
	Epithelioid sarcoma (1)	Nasopharyngeal carcinoma (1)	2
	Leiomyosarcoma (1)	Squamous cell carcinoma (1)	2
	Malignant hemangioendothelioma		1
	Mucoepidermoid carcinoma		1
Benign			
	Granuloma pygenicum (5)	Hemangioma (2)	7
	Benign fibrous histiocytoma (1)	Lipoma (1)	2
	Giant cell tumor		1
	Hemangioma		2
	Neurofibroma		2
	Pseudo epitheliomatous hyperplasia		1
	Lentigo		1
Inflammatory			
	Eosinophilic pustular folliculitis (1)	Lymphogranuloma inguinale (1)	2
	Histoplasmosis (1)	Tuberculous adenitis (1)	2
	Maduro mycosis		1
	Granulation tissue (3)		4
	Panniculitis (1)		2
	Stasis dermatitis		2
	Tuberculosis		1
	Verrucous vulgaris		1
	Vasculitis		2

**Table 3.** Histopathological KS Diagnosis Discordance before HHV-8-LANA-1 IHC Confirmation

	False positive	False-negative	(N=15)
Malignant	Leiomyosarcoma		1
	Dermatofibrosarcoma protuberans		1
Benign			
	Granuloma pyogenicum (3)	Granuloma pyogenicum (1)	4
	Benign fibrous histiocytoma		1
	Hemangioma		2
Inflammatory			
	Vasculitis (1)	Chronic inflammation-NOS (1)	2
	Stasis dermatitis (2)	Granulation tissue (1)	3
	Panniculitis (1)		1

(A) H&E at 40x, (B) H&E at 100x, (C) Positive HHV-8-LANA-1 IHC at 40x and (D) Positive HHV-8-LANA-1 IHC at 100x.

(A) H&E at 40x, (B) H&E at 100x, (C) Positive HHV-8-LANA-1 IHC at 40x and (D) Positive HHV-8-LANA-1 IHC at 100x

## DISCUSSION

Among all confirmed KS, HIV negative was 10.2% thus classified as Endemic African Endemic KS, which was higher than reported by Amos R Mwakigonja et al MNH in 2007^9^ but slightly lower than that reported by Wamburu et al at Kilimanjaro Christian Medical Center (KCMC).^3^ This difference could be due to improvement in current HIV/AIDS transmission prevention programs. Nevertheless, due to the high incidence of HIV in Africa, it poses a challenge for both clinical and histopathological to distinguish Endemic African KS and AIDS-related KS in the people living with HIV (PLWH).^3^ The clinical and demographic characteristics of KS are variable.^26^ There was a wide age range from children to elderly, which implies that KS can occur at any age. The median age was similar to reported in a local study at ORCI,^27^ and the median duration of symptoms was 6 months which was similar to Kamyab et al and Chalya et al studies.^7,28^

Complete and accurate clinical history including HIV status is important for histopathological diagnosis of KS. In our study significant number of patients’ HIV status was not reported and even among people living with HIV (PLWH), a similar high rate of under-reporting of HIV status was showed by Louis-Jacque van Bogaert in South Africa, was comparably higher than our findings.^12^ This study also has demonstrated one case of nasopharyngeal KS, which is extremely rare.^29^ This patient was 32 years old female, HIV seroconverted, on ART with nasopharynx mass extending to the nasal cavity.

Despite the clinical relevance of KS in Tanzania, little is known about the precision of its clinical suspicion. Our current study showed no agreement between clinical and HHV-8-LANA-1 IHC for KS diagnosis with a relatively low concordance rate and positive predictive rate, which were lower than reported in South Africa,^12^ and in studies done in Kenya and Uganda.^10^ This dissimilarity may be due to difference in a number of experienced clinicians in different countries. In terms of good clinical practice, this suggests that reliance on visual impressions alone needs to be stopped. When KS was not a correct clinical diagnosis, differential diagnoses ranged from self-limiting inflammatory conditions to fatal malignant conditions with different management and required appropriate distinction.

Currently, in Tanzania there is good progress in the availability of histopathology services, however, in most of the anatomical pathology departments, KS diagnosis is done by routine H&E histopathology alone. Almost perfect agreement between initial and reviewed H&E histopathological diagnosis of KS indicates that misdiagnosis is unlikely to be due to human error. To the best of our knowledge, this is the first study done in Tanzania to assess the correctness of the histopathology diagnosis of KS. Also, our current study showed substantial agreement between initial histopathology and HHV-8-LANA-1 IHC with a high concordance rate and positive predicted value. This agreement was higher compared to the study done in Uganda and Kenya, ^10^ but this was slightly lower than a study done in South Africa,^12^ the differences could be due to different level of histopathology subspecialties among countries.

**Figure 3.**
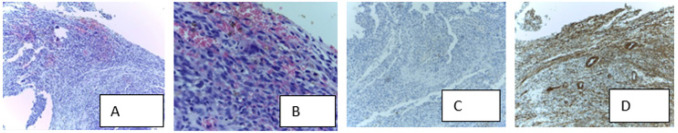
Discordant Pair of Leiomyosarcoma Cases which was Diagnosed as Kaposi’s Sarcoma by Histopathology before HHV-8-LANA-1 IHC

**Figure 4.**
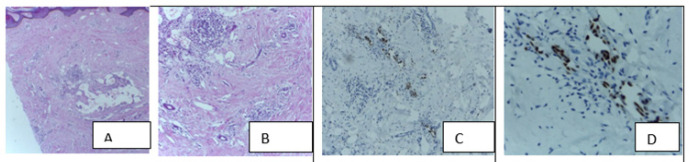
Early Kaposi’s Sarcoma with Inconspicuous Histopathological Features which was Diagnosed as Chronic Inflammation NOS before HHV-8-LANA-1 IHC.

The diagnostic difficulty was common in differentiating granuloma pyogenicum from KS by histomorphology. This diagnostic challenge was also reported in other studies.^10–12,22^ One case with histomorphology features of KS was a leiomyosarcoma as it was negative for HHV-8-LANA-1 IHC but positive to smooth muscle actin IHC, this implicates consideration of non-vascular soft tissue lesions as KS histopathological differential diagnosis (Figure 3). Furthermore, three cases with histomorphology features of chronic inflammation NOS (Figure 4), granulation tissue, and panniculitis, respectively were positive for HHV-8-LANA-1 IHC. This demonstrates that histopathological features of KS may be missed in the early stage, and these are cases that may require routine HHV-8-LANA-1 IHC stain as pointed out by Radu et al and Grayson W.^15,16^

(A) H&E at 100x, (B) H&E at 400x, (C) Negative HHV-8-LANA IHC at 100x and (D) Smooth muscle actin IHC at 100x

(A) H&E at 100x, (B) H&E at 400x, (C) Negative HHV-8-LANA IHC at 100x and (D) Smooth muscle actin IHC at 100x

### Study limitations

Being a retrospective study relevant clinical information was missing in a significant number of cases which might have affected the association between clinical features and KS and other information such as CD4+ counts could not be assessed.

## CONCLUSION AND RECOMMENDATIONS

Histopathological examination of all clinical KS suspicions and HHV-8-LANA-1 immunohistochemistry confirmation are required since the study showed that the histopathology misdiagnosis of KS at MNH was unlikely to be the result of human error.

We recommend that in every clinically suspected KS case, an adequate tissue biopsy should be taken for histopathology analysis and HHV8-LANA-1 immunostaining to avoid giving inappropriate treatment.
